# Effect of Antenatal Monthly Sulfadoxine–Pyrimethamine, Alone or with Azithromycin, on Gestational Weight Gain and Anemia during Pregnancy and One Month Postpartum in Malawi: A Randomized Controlled Trial Secondary Analysis

**DOI:** 10.4269/ajtmh.23-0829

**Published:** 2025-02-04

**Authors:** Mari Luntamo, Lotta Hallamaa, Teija Kulmala, Kenneth Maleta, Per Ashorn

**Affiliations:** ^1^ Faculty of Medicine and Health Technology, Center for Child, Adolescent, and Maternal Health Research, Tampere University, Tampere, Finland;; ^2^ Department of Nutrition and Dietetics, School of Global and Public Health, Kamuzu University of Health Sciences, Blantyre, Malawi;; ^3^ Department of Pediatrics, Tampere University Hospital, Tampere, Finland

## Abstract

Low gestational weight gain (GWG) and prenatal anemia are associated with adverse pregnancy, maternal and infant health outcomes. In a secondary analysis of a single-center, randomized, partially placebo-controlled, outcome assessor-blinded, controlled trial conducted in Malawi from 2003 to 2006, when antiretroviral treatment (ART) for HIV was not widely available, we studied whether GWG can be increased and the prevalence of maternal anemia decreased during pregnancy and at 1 month postpartum through the intermittent preventive treatment in pregnancy (IPTp) of maternal malaria and reproductive tract infections. The participants (≥15-year-old women with uncomplicated second trimester single pregnancies) received either sulfadoxine (1,500 mg) and pyrimethamine (75 mg; SP) twice (control group, *n* = 433), monthly SP (*n* = 439), or monthly SP and azithromycin (1,000 mg) twice (AZI-SP, *n* = 441) during pregnancy. The mean weekly GWG in the sample was 256 g/week. The participants in the monthly SP group gained, on average (95% CI), 4 g (–13 to 20; *P* = 0.671), and those in the AZI-SP group gained 25 g (8–41; *P* = 0.003) more weight per week than control group participants. Among HIV-positive participants (12%), the differences were larger and also significant between the monthly SP group and control group. Mean hemoglobin and anemia prevalence did not differ between the groups during pregnancy or postnatally. The data support a hypothesis that IPTp with monthly SP and two doses of azithromycin can increase GWG, especially among HIV-positive women who are not on ART, possibly through the reduction of infections, inflammation, and effects on the maternal gut microbiome.

## INTRODUCTION

Macro- and micronutrient undernutrition during pregnancy is common in low- and middle-income countries and is associated with adverse pregnancy, maternal and infant health outcomes.^1^^–^^3^ In 2019 in eastern Africa, 10% of women 18 years and older were underweight (defined as body mass index [BMI] <18.5 kg/m^2^), and 38% of pregnant women aged 15–49 had anemia (defined as hemoglobin [Hb] <110 g/l).^4^ Low gestational weight gain (GWG) and anemia during pregnancy, which are manifestations of maternal macro- and micronutrient undernutrition, respectively, have been associated with a higher risk of preterm birth, small for gestational age (SGA) at delivery, and low birth weight (LBW).^5^^–^^7^ Preterm birth, SGA, and LBW are risk factors for neonatal and postneonatal mortality and negative long-term outcomes.^8^^–^^12^ A modeling analysis estimated that in 2015 women in sub-Saharan Africa met less than 60% of the minimum GWG recommendation for normal-weight women (11.5 kg weight gain during pregnancy).^13^

Maternal nutritional supplementation has been shown to provide some benefits for mothers and their offspring.^14^^–^^17^ However, maternal infections may also contribute to low GWG and anemia, for example, by causing malabsorption and increasing energy consumption due to immune responses, thus increasing the risk of and exacerbating existing undernutrition.^18^^–^^20^ Some infections, such as malaria, also directly cause anemia.^18^^–^^21^ Furthermore, malaria is independently associated with adverse pregnancy outcomes and maternal and neonatal morbidity and mortality.^22^^–^^24^ Therefore, in addition to improving maternal nutrition, the prevention and treatment of gestational infections is essential, especially in settings with a high prevalence of these infections.

Intermittent preventive treatment in pregnancy (IPTp) against malaria with three or more doses of sulfadoxine–pyrimethamine (SP) has been shown to reduce the risk of moderate to severe anemia among paucigravid women.^25^ However, it is uncertain whether infection control during pregnancy affects GWG. A study conducted in Papua New Guinea (PNG) found that pregnant women who received three courses of SP and azithromycin, which is a wide-spectrum antibiotic that is effective against many reproductive tract infections (RTIs) and has modest antimalarial activity,^26^^–^^27^ had higher GWG than women who received a single treatment course of SP and chloroquine at first antenatal visit.^28^ The prevalence of anemia at delivery was similar in the study groups.^29^ The group that received azithromycin also had a lower prevalence of preterm delivery, LBW, and maternal malaria at delivery.^29^

We have previously reported the results of a three-arm randomized controlled trial that tested whether the intensive prevention and treatment of malaria and RTIs in rural Malawi result in improved pregnancy outcomes and maternal and infant health.^30^^–^^32^ In this trial, participants who received IPTp with monthly SP and two doses of azithromycin, instead of two doses of SP, which was standard care at the time of the study, had a lower prevalence of maternal infections, preterm delivery, and LBW, and their infants had less stunting and underweight at 1 month of age.^30^^–^^32^ Intermittent preventive treatment in pregnancy with monthly SP and without azithromycin yielded fewer benefits.^30^^–^^32^ In this study, we report the effect of these interventions on maternal weight gain and anemia during pregnancy and 1 month after delivery.

## MATERIALS AND METHODS

### Study design.

We undertook a single-center, randomized, partially placebo-controlled, outcome assessor-blinded, three-arm clinical trial in rural Malawi. The study hypothesis was that adverse pregnancy outcomes can be reduced and maternal and infant health can be improved by IPTp with monthly SP, alone or in combination with two doses of azithromycin. The primary efficacy and safety outcomes, namely, the incidence of preterm delivery and serious adverse events, as well as other details of the study, have been reported previously.^30^ In the current study, we report the results of a secondary analysis on predefined secondary outcomes regarding maternal anthropometrics and anemia during pregnancy and 1 month after delivery.

### Participants and study site.

The study participants were 15-year-old or older women with uncomplicated second trimester single pregnancies (gestational age 14–26 weeks by ultrasound assessment) who started antenatal care between December 2003 and October 2006 at Lungwena Health Centre, southern Malawi. Malaria is holoendemic at the study site, and earlier evidence suggested a high prevalence of RTIs among pregnant women.^33^^–^^34^ Only participants who signed or thumb-printed an informed consent form were enrolled in the study. Key details of the protocol were published in the clinical trial registry of the National Library of Medicine (Bethesda, MD; http://www.clinicaltrials.gov, trial identification NCT00131235).

### Study interventions.

Participants in the control group received standard Malawian antenatal care, which at the time of the study included IPTp with two doses of SP (three tablets per os, each containing 500 mg of sulfadoxine and 25 mg of pyrimethamine); they received the first dose at enrollment and the second between 28 and 34 gestational weeks (gw). Participants in the monthly SP and azithromycin–SP (AZI-SP) intervention groups received the same amount of SP monthly (with a minimum of 4 weeks between the doses) from enrollment until 37 gw. At enrollment and between 28 and 34 gw, participants in the AZI-SP intervention group received a dose of active azithromycin (two tablets per os, each containing 500 mg of azithromycin), whereas the participants in the other two groups received two placebo tablets. All participants received ferrous sulfate (200 mg/day) and folic acid (0.25 mg/day) throughout pregnancy. Sulfadoxine–pyrimethamine tablets were purchased from Malawi Central Medical Stores, and active azithromycin and its placebo were manufactured and donated by Pfizer Inc. (New York, NY).

### Enrollment.

At enrollment, research personnel interviewed interested individuals about their socioeconomic status, living conditions, disease and obstetric history, provided pre-test HIV counseling, and performed an antenatal examination. Participants’ weight with light clothes was measured with electronic standing scales (OBH Nordica, Taastrup, Denmark), and the results were recorded to the nearest 100 g. The scales were calibrated with standard weights once a week. A laboratory assistant assessed the Hb concentration of all participants with a HemoCue machine (HemoCue AB, Ängelholm, Sweden). Its correct functioning was checked each measurement day with a control cuvette and once a week by comparing the analysis results of two HemoCue machines. Participants’ mid-upper arm circumference (MUAC) was measured from the left arm, midway between the tip of the acromion and the olecranon process, with a nonstretchable plastic Lasso-o tape (Harlow Printing Limited, South Shields, United Kingdom; reading increment 1 mm), and their height was measured with a portable Harpenden stadiometer (Holtain Limited, Crymych, United Kingdom; reading increment 1 mm).

A midwife determined the duration of pregnancy with an ultrasound imager. Standard methods described previously were used to assess the microscopic peripheral blood malaria parasitemia and syphilis status of all participants and to perform HIV tests for those who opted for them.^30^ HIV test results were categorized as positive, negative, and unknown (the last category included both those who did not opt for an HIV test and those with indeterminate results). Participants with confirmed syphilis were treated with intramuscular benzathine penicillin (2.4 million units), and their infants received the same drug (50,000 units/kg) at the age of 4 weeks.

Individuals who were eligible and willing to participate signed or thumb-printed an informed consent form and picked one randomization envelope that contained an identification number (for details on randomization, see the previous publication).^30^ A research assistant not involved in the outcome assessment provided the corresponding prepackaged study drugs to the participant under direct observation and monitored her for possible adverse reactions.

### Follow-up.

At follow-up visits (at 4-week intervals until 36 completed gw and weekly thereafter), research personnel conducted an antenatal examination, including weight, Hb, and MUAC measurements, as described above. The participants were asked about health problems, including gastrointestinal symptoms. A midwife examined participants’ faces, hands, and legs for edema. The participants were offered post-test HIV counseling and those who were diagnosed to be living with HIV received nevirapine for prevention of mother-to-child transmission. Other antiretroviral treatment (ART) was not available at the time of the study. At each visit, participants took the appropriate prepackaged study drugs under direct observation. At the postnatal visit ∼1 month after delivery, research personnel measured participants’ weight, Hb, and MUAC.

## STATISTICAL ANALYSES

The original sample size of 1,320 pregnant women was estimated to have 80% power at a 5% level of significance to detect a 40% reduction in the primary efficacy outcome (rate of preterm delivery, defined as birth before 37 completed gw) between the AZI-SP and the control group.^30^ Statistical analyses were conducted with Stata 9.2 and Stata 15 (StataCorp, College Station, TX). Each participant’s trajectories of weight, Hb, and MUAC during pregnancy were examined with a mixed-effects model, and implausible values were excluded. The original analysis plan included comparisons of the two intervention groups (monthly SP and AZI-SP) with the control group that received SP twice, which was standard treatment at the time the study was implemented. The study was not powered to test formal hypotheses between the monthly SP and AZI-SP groups. However, because of changes in the global IPTp guidelines after the study was implemented, which now recommend at least three doses of IPTp-SP during pregnancy as the standard of care,^35^ additional analyses comparing the results between the monthly SP and AZI-SP groups were included in this manuscript.

A linear mixed-effects model was used to estimate GWG in the three study groups. All participants with at least one weight measurement at enrollment or during pregnancy follow-up were included in the analyses. The mixed-effects model adjusts for the effect of missing values by using the correlation between repeated measurements and can thus be used to estimate parameters without imputing the missing data, even if only one measurement exists.^36^ The model included random intercepts by participant and a random slope by the duration of pregnancy, and the variance–covariance structure of the random effects was allowed to be correlated. We stored the estimates in a new variable, calculated group means for GWG, and used least squares regression to estimate differences between the groups.

Because of a nonlinear trend during pregnancy, maternal Hb and MUAC were analyzed at 28.00–33.99 gw, when the participants received the second dose of azithromycin or placebo. If a participant had more than one measurement within these gw, the latest value was used. Any, moderate, or severe anemia during pregnancy were defined as at least one Hb measurement below 110 g/l, 80 g/l, and 50 g/l, respectively, at any follow-up visit after enrollment but before delivery. The same definitions of anemia were used for the postnatal values. For Hb and MUAC during pregnancy and postnatally and for postnatal weight and BMI, we calculated group means and used least squares regression to estimate the differences between the groups. For the prevalence of anemia, we calculated percentages and used a log-binomial regression model to estimate RRs. Postnatal variables with a measuring date that differed more than 2 weeks from the target of 4 weeks after delivery were excluded from the analyses. Means and RRs (except for the RR of anemia during pregnancy) were adjusted for the respective enrollment value and timing of measurement.

To prevent inflated type I errors due to testing between multiple groups, we began hypothesis testing with the global null hypothesis of all three groups being identical. For pairwise comparisons, the hypothesis of no difference between the groups was rejected only if the global null hypothesis was also rejected. All *P*-values were two-sided, and the null hypothesis was rejected if the *P*-value was less than 0.05.

The proportion of adolescents, primi- and secundigravida, and participants with malaria parasitemia at enrollment was higher in the control group than in the intervention groups. We performed sensitivity analyses adjusting the continuous variables for these three covariates as categorical variables. We included 48 individuals who participated in the trial twice with consecutive pregnancies in the analyses. Due to this clustering of participants, we performed sensitivity analyses using robust standard errors, which allowed for intragroup correlation. Because edema and chronic diarrhea during pregnancy can affect GWG, we analyzed whether there were differences between the groups in these variables.

As exploratory analyses, we performed tests for interactions for GWG between the interventions and HIV status, number of previous pregnancies, bed net use, adolescence, maternal BMI category, short stature (height less than 150 cm), low MUAC (<23 cm), any anemia, and literacy at enrollment. The interaction tests were performed using Wald’s test. If a statistically significant interaction at *P* <0.1 was found between the interventions and one of the variables, we performed analyses stratified by that variable. Irrespective of the result of the interaction test, we performed analyses stratified by the following variables: HIV status, number of previous pregnancies, adolescence at enrollment, and bed net use the night before enrollment. As sensitivity analyses, the stratified analyses were adjusted for all the other stratification variables (HIV infection, gravidity, adolescence, and bed net use), and the results were compared with the unadjusted stratified analysis results.

## RESULTS

**Figure 1.**
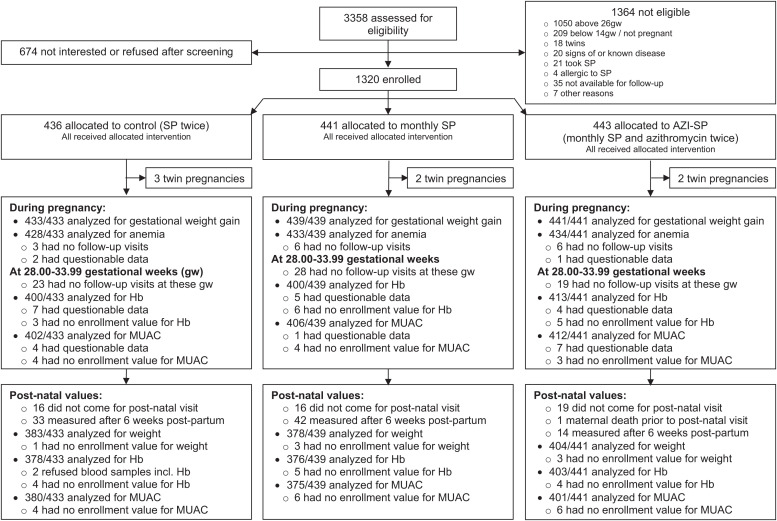
Participant flow in Consolidated Standards of Reporting Trials (CONSORT) recommended format.

Of the 3,358 pregnant women we invited to participate in the study, 1,320 were randomly assigned to the three intervention groups, as follows: control, monthly SP, and AZI-SP (Figure 1). The enrolled individuals and those not enrolled had approximately the same mean age (25 versus 26 years, respectively) and number of previous pregnancies (2.3 versus 2.5, respectively). Seven inadvertently enrolled women with twin pregnancies were excluded from the current analyses, thus making the total number of included participants 1,313. At enrollment, the three groups had similar baseline characteristics, except for small differences in the proportion of adolescent participants (age 15–19), number of previous pregnancies, and prevalence of malaria parasitemia (Table 1). The prevalence of malaria at enrollment was highest in the control group (11%) and lowest in the AZI-SP group (6%). Of all participants, 161 (12%) were HIV-positive, 1,028 (78%) were HIV-negative, and 124 (9%) had unknown HIV status, with no major differences between the study groups.

**Table 1:** Baseline characteristics of participants at enrollment

Characteristic	Control (SP twice)	Monthly SP	AZI-SP
Number of enrolled women	433	439	441
Mean (SD) age in years	25 (7)	25 (7)	25 (6)
Proportion (%) of adolescent participants (age 15–19)	120/433 (27.7)	109/439 (24.8)	83/441 (18.8)
Mean (SD) years of schooling completed	2.1 (2.7)	2.1 (2.6)	2.4 (2.8)
Proportion (%) of literate participants	115/433 (26.6)	128/439 (29.2)	138/441 (31.3)
Proportion (%) with a safe source of drinking water (defined as piped or borehole water)	371/433 (85.7)	378/439 (86.1)	369/441 (83.7)
Proportion (%) with adequate sanitation (defined as the availability of a pit latrine)	406/433 (93.8)	411/439 (93.6)	416/441 (94.3)
Proportion (%) with chronic diarrhea	1/431 (0.2)	0/438 (0.0)	3/441 (0.7)
Mean (SD) gestational age in weeks	20.3 (3.0)	20.0 (3.2)	20.0 (3.0)
Mean (SD) height in cm	155.0 (5.6)	154.8 (5.4)	155.3 (5.5)
Mean (SD) weight in kg	52.2 (6.1)	52.3 (6.4)	52.9 (6.2)
Mean (SD) BMI in kg/m^2^	21.7 (2.2)	21.8 (2.1)	21.9 (2.1)
BMI category, proportion (%)			
Underweight (BMI <18.5)	24/432 (5.6)	19/436 (4.4)	20/436 (4.6)
Normal (18.5 ≤ BMI <25)	379/432 (87.7)	380/436 (87.2)	387/436 (88.8)
Overweight (BMI ≥25)	29/432 (6.7)	37/436 (8.5)	29/436 (6.7)
Mean (SD) mid-upper arm circumference in cm	25.1 (2.0)	25.2 (2.1)	25.3 (1.8)
Mean (SD) Hb concentration as g/l	110 (19)	111 (16)	110 (19)
Anemia status, proportion (%)			
Any anemia (Hb <110 g/l)	211/429 (49.2)	201/433 (46.4)	211/436 (48.4)
Moderate anemia (Hb <80 g/l)	21/429 (4.9)	8/433 (1.9)	27/436 (6.2)
Proportion (%) with microscopic peripheral blood malaria parasitemia	48/432 (11.1)	41/439 (9.3)	27/441 (6.1)
Proportion (%) who used bed net during the previous night	267/433 (61.7)	261/439 (59.5)	266/441 (60.3)
Number of previous pregnancies, proportion (%)			
None	108/433 (24.9)	107/439 (24.4)	88/441 (20.0)
One	85/433 (19.6)	76/439 (17.3)	81/441 (18.4)
Two or more	240/433 (55.4)	256/439 (58.3)	272/441 (61.7)
HIV status, proportion (%)			
Positive	48/433 (11.1)	64/439 (14.6)	49/441 (11.1)
Negative	345/433 (79.7)	335/439 (76.3)	348/441 (78.9)
Unknown	40/433 (9.2)	40/439 (9.1)	44/441 (10.0)
Syphilis status, proportion (%)			
Positive	18/433 (4.2)	27/439 (6.2)	21/441 (4.8)
Negative	412/433 (95.2)	406/439 (92.5)	417/441 (94.6)
Unknown	3/433 (0.7)	6/439 (1.4)	3/441 (0.7)

AZI-SP = azithromycin and sulfadoxine–pyrimethamine; BMI = body mass index; Hb = hemoglobin; SP = sulfadoxine–pyrimethamine.

The mean (SD) number of scheduled SP doses received was 2.0 (0.2) in the control, 4.0 (1.0) in the monthly SP, and 4.0 (0.9) in the AZI-SP group. Women in the AZI-SP group received a mean (SD) of 2.0 (0.2) azithromycin doses. Against the trial protocol, some SP doses were given in error by the health facility personnel at unscheduled visits (mean 0.008 doses/participant; *P* = 0.310 for intergroup difference).

Data on gestational weight during enrollment or pregnancy follow-up were available for all participants, data on anemia during pregnancy were available for 99% of participants, and data for an analysis of Hb and MUAC at 28.00–33.99 gw adjusted for the timing of measurement and enrollment value were available for 92% and 93% of participants, respectively (Figure 1). There were no differences between the groups in the mean number of measurements of maternal weight (4.3–4.4; *P* = 0.482 for intergroup difference) or the mean duration of pregnancy at the first (20.1–20.3 gw; *P* = 0.472) and last (35.2–35.7 gw; *P* = 0.105) weight measurements. Slightly more participants in the AZI-SP group (91–92%) had adjusted analyses available for postnatal weight, Hb, and MUAC than the participants in the control (87–88%) or monthly SP (85–86%) groups (*P* = 0.023–0.040 for intergroup differences) due to the exclusion of more measurements in the control and monthly SP groups because of late timing of the 1-month postnatal visit (Figure 1). The background characteristics of the participants who had postnatal measurements within 2 weeks of the intended measurement date and those who did not, were otherwise similar, except that those who had measurements within the allowed timeframe attended the enrollment visit slightly earlier (20.0 gw) than those who were measured after 6 weeks postpartum (21.5 gw; data not shown).

**Figure 2.**
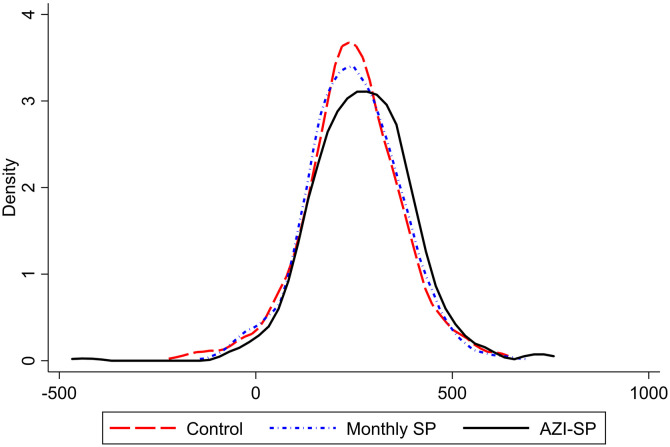
Distribution of gestational weight gain (g/week).

The overall mean (SD) weekly GWG was 256 g (12). Figure 2 shows the GWG distribution by treatment group. The participants in the monthly SP group gained, on average (95% CI), 4 g (–13 to 20; *P* = 0.671; global *P* = 0.006), and those in the AZI-SP group gained 25 g (8–41; *P* = 0.003) more weight per week than control group participants (Table 2). When comparing the two intervention groups, the participants in the AZI-SP group gained, on average (95% CI), 21 g (5–37; *P* = 0.011) more weight per week than the monthly SP group participants. A sensitivity analysis adjusting for baseline malaria, gravidity, and adolescence produced similar results.

**Table 2:** Gestational weight gain during pregnancy

Outcome	Mean (SD) Result by Study Group				Comparison Between Monthly SP and Control Group		Comparison Between AZI-SP and Control Group		Comparison Between AZI-SP and Monthly SP Group	
	Control	Monthly SP	AZI-SP	*P*-Value	Mean Difference (95% CI)	*P*-Value	Mean Difference (95% CI)	*P*-Value	Mean Difference (95% CI)	*P*-Value
Mean (SD) GWG (g/week) from enrollment to delivery										
Unadjusted	246 (122)	250 (118)	271 (126)	0.006	4 (−13 to 20)	0.671	25 (8 to 41)	0.003	21 (5 to 37)	0.011
Adjusted for malaria, gravidity, and adolescence at enrollment	247 (122)	250 (118)	271 (126)	0.007	3 (−13 to 20)	0.684	24 (8 to 40)	0.004	21 (5 to 37)	0.012
Mean (SD) GWG (g/week) from enrollment to delivery (unadjusted), stratified by HIV status at enrollment										
Negative (*n* = 1,028)	257 (119)	251 (119)	274 (124)	0.034	−6 (−24 to 12)	0.540	17 (−1 to 35)	0.059	23 (5 to 41)	0.013
Positive (*n* = 161)	161 (115)	239 (121)	281 (151)	0.000	78 (29 to 126)	0.002	120 (68 to 172)	0.000	42 (−6 to 91)	0.086
Unknown (*n* = 124)	254 (128)	254 (95)	233 (110)	0.600	1 (−49 to 50)	0.983	−21 (−69 to 27)	0.392	−22 (−70 to 27)	0.380
Negative and unknown combined (*n* = 1,152)	257 (120)	252 (117)	270 (122)	0.101	−5 (−22 to 12)	0.562	13 (−4 to 30)	0.134	18 (1 to 35)	0.039
Mean (SD) GWG (g/week) from enrollment to delivery (unadjusted), stratified by the number of previous pregnancies at enrollment										
None (*n* = 303)	231 (122)	243 (120)	254 (129)	0.456	11 (−22 to 45)	0.496	22 (−13 to 57)	0.212	11 (−24 to 46)	0.548
One (*n* = 242)	257 (111)	245 (98)	243 (110)	0.654	−12 (−46 to 21)	0.469	−14 (−47 to 19)	0.397	−2 (−36 to 32)	0.914
Two or more (*n* = 768)	249 (126)	254 (122)	285 (129)	0.002	6 (−17 to 28)	0.618	36 (14 to 58)	0.001	30 (9 to 52)	0.006
Mean (SD) GWG (g/week) from enrollment to delivery (unadjusted), stratified by adolescence at enrollment										
No (adult) (*n* = 1,001)	249 (127)	252 (122)	279 (126)	0.003	3 (−16 to 22)	0.768	29 (10 to 48)	0.003	26 (8 to 45)	0.006
Yes (*n* = 312)	238 (107)	242 (104)	235 (122)	0.905	4 (−25 to 33)	0.775	−3 (−34 to 28)	0.855	−7 (−39 to 25)	0.661
Mean (SD) GWG (g/week) from enrollment to delivery (unadjusted), stratified by bed net use the night before enrollment										
No (*n* = 519)	222 (110)	210 (109)	241 (118)	0.033	−12 (−36 to 12)	0.336	19 (−5 to 43)	0.113	31 (8 to 55)	0.010
Yes (*n* = 794)	261 (126)	277 (117)	291 (128)	0.024	16 (−6 to 37)	0.149	29 (8 to 50)	0.006	14 (−7 to 35)	0.203

AZI-SP = azithromycin and sulfadoxine–pyrimethamine; GWG = gestational weight gain; SP = sulfadoxine–pyrimethamine.

There was an interaction between GWG and HIV status at enrollment (*P* = 0.019), but not with the other tested variables (*P* >0.1). The exploratory stratified analyses suggested that compared with the control group participants, those who were HIV-positive, multigravida, 20 years of age or older, or used a bed net at enrollment had higher weekly GWG in the AZI-SP group, whereas only those who had HIV infection gained more weight in the monthly SP group (Table 2). When compared with the monthly SP group participants, the participants in the AZI-SP group who were HIV-negative, multigravida, 20 years of age or older, or did not use a bed net at enrollment had higher weekly GWG. Among HIV-positive participants, those in the AZI-SP group gained, on average (95% CI), 120 g (68–172; *P* = 0.000), and those in the monthly SP group gained 78 g (29–126; *P* = 0.002) more weight per week than participants in the control group, whereas among HIV-negative participants, those in the AZI-SP group gained, on average (95% CI), 23 g (5–41; *P* = 0.013) more weight per week than participants in the monthly SP group. There were no differences between the other groups. Stratified analyses adjusted for all the stratification variables (HIV infection, gravidity, adolescence, and bed net use) yielded almost identical results to the unadjusted stratified analyses (data not shown).

Among all participants, mean (SD) Hb was 111 g/l (13) at 28.00–33.99 gw and 124 g/l (16) at 1 month after delivery. The mean difference (95% CI) in Hb between the AZI-SP and the control group was 2 g/l (0–4, *P* = 0.017; global *P* = 0.058) at 28.00–33.99 gw in the main analysis, adjusted for the timing of measurement and Hb at enrollment, and 2 g/l (1–4, *P* = 0.011; global *P* = 0.040) in the analysis additionally adjusted for malaria, gravidity, and adolescence at enrollment (Table 3). There were no differences between the control and monthly SP groups, or between the monthly SP and AZI-SP groups, in Hb during pregnancy, and no differences in Hb were observed between any of the groups at postnatal visits.

**Table 3:** Maternal hemoglobin at 28.00–33.99 gestational weeks of pregnancy, anemia during pregnancy, and hemoglobin and anemia at 1 month after delivery

Outcome	Mean (SD) Result or Number of Outcome/Participants With Outcome Data (%)				Comparison Between Monthly SP and Control Group		Comparison Between AZI-SP and Control Group		Comparison Between AZI-SP and Monthly SP Group	
	Control	Monthly SP	AZI-SP	*P*-Value	Mean Difference or RR (95% CI)	*P*-Value	Mean Difference or RR (95% CI)	*P*-Value	Mean Difference or RR (95% CI)	*P*-Value
Mean (SD) Hb (g/l) at 28.00–33.99 gw										
Adjusted for the timing of measurement and Hb at enrollment	110 (12)	111 (13)	112 (13)	0.058	1 (−1 to 3)	0.290	2 (0 to 4)	0.017	1 (−1 to 3)	0.189
Adjusted for the timing of measurement and Hb, malaria, gravidity, and adolescence at enrollment	110 (12)	111 (13)	112 (13)	0.040	1 (−1 to 3)	0.290	2 (1 to 4)	0.011	1 (0 to 3)	0.141
Mean (SD) maternal Hb (g/l) at 1 month after delivery										
Adjusted for the timing of measurement and Hb at enrollment	123 (15)	124 (17)	125 (16)	0.236	1 (−2 to 3)	0.511	2 (0 to 4)	0.092	1 (−1 to 3)	0.310
Adjusted for the timing of measurement and Hb, malaria, gravidity, and adolescence at enrollment	123 (15)	124 (17)	125 (16)	0.195	1 (−2 to 3)	0.545	2 (0 to 4)	0.076	1 (−1 to 4)	0.244
Maternal anemia at any time after enrollment and before delivery*										
Any anemia (Hb <110 g/l)	298/428 (69.6)	286/433 (66.1)	284/434 (65.4)	0.356	0.95 (0.87 to 1.04)	0.262	0.94 (0.86 to 1.03)	0.190	0.99 (0.90 to 1.09)	0.849
Moderate (Hb <80 g/l) or severe^†^ (Hb <50 g/l) anemia	23/428 (5.4)	17/433 (3.9)	21/434 (4.8)	0.601	0.73 (0.40 to 1.35)	0.315	0.90 (0.51 to 1.60)	0.721	1.23 (0.66 to 2.30)	0.512
Maternal anemia at 1 month after delivery*^‡^										
Any anemia (Hb <110 g/l)	74/382 (19.4)	57/381 (15.0)	70/407 (17.2)	0.223	0.76 (0.56 to 1.04)	0.084	0.88 (0.66 to 1.17)	0.389	1.15 (0.85 to 1.58)	0.366
Moderate (Hb <80 g/l) or severe^§^ (Hb <50 g/l) anemia	6/382 (1.6)	10/381 (2.6)	9/407 (2.2)	0.186	2.32 (0.86 to 6.22)	0.095	1.20 (0.45 to 3.17)	0.719	0.52 (0.21 to 1.26)	0.147

AZI-SP = azithromycin and sulfadoxine–pyrimethamine; gw = gestational weeks; Hb = hemoglobin; SP = sulfadoxine–pyrimethamine.

* For anemia, unadjusted number of observations and proportions.

^†^ Only two participants had severe anemia: one in the monthly SP group and one in the AZI-SP group.

^‡^ For anemia 1 month after delivery, *P*-values, risk ratios, and CIs are adjusted for the timing of measurement and any anemia/moderate or severe anemia at enrollment.

^§^ No participant had severe anemia.

Of all participants, 67% had any anemia, 4.7% had moderate anemia, and 0.2% had severe anemia at least once during pregnancy follow-up. One month after delivery, the prevalence was 17% for any anemia, 2.1% for moderate anemia, and 0.0% for severe anemia. There were no differences between the groups in the prevalence of anemia at either time point (Table 3).

Mean (SD) MUAC among all participants was 25.1 cm (1.0) at 28.00–33.99 gw and 24.9 cm (1.3) at ∼4 weeks after delivery. The mean difference (95% CI) in MUAC between the AZI-SP and the control group was 0.2 cm (0.0–0.3; *P* = 0.032; global *P* = 0.094) during pregnancy and 0.2 cm (0.0–0.4; *P* = 0.015; global *P* = 0.046) at the postnatal visit (Table 4). There were no differences between the control and monthly SP groups or between the monthly SP and AZI-SP groups in MUAC during pregnancy or the postnatal visit. At ∼1 month after delivery, the mean (SD) maternal weight and BMI among all participants were 51.2 kg (2.9) and 21.2 kg/m^2^ (1.2), respectively. There were no differences between the groups in these anthropometric measurements.

**Table 4:** Maternal mid-upper arm circumference at 28.00–33.99 gestational weeks and maternal mid-upper arm circumference, weight, and BMI at 1 month after delivery

Outcome	Mean (SD) Result by Study Group				Comparison Between Monthly SP and Control Group		Comparison Between AZI-SP and Control Group		Comparison Between AZI-SP and Monthly SP Group	
	Control	Monthly SP	AZI-SP	*P*-Value	Mean Difference (95% CI)	*P*-Value	Mean Difference (95% CI)	*P*-Value	Mean Difference (95% CI)	*P*-Value
Mean (SD) MUAC (cm) at 28.00–33.99 gw										
Adjusted for the timing of measurement and MUAC at enrollment	25.0 (1.0)	25.1 (1.0)	25.2 (1.0)	0.094	0.1 (−0.1 to 0.2)	0.437	0.2 (0.0 to 0.3)	0.032	0.1 (0.0 to 0.2)	0.170
Adjusted for the timing of measurement and MUAC, malaria, gravidity, and adolescence at enrollment	25.0 (1.0)	25.1 (1.0)	25.1 (1.0)	0.163	0.0 (−0.1 to 0.2)	0.518	0.1 (0.0 to 0.3)	0.061	0.1 (−0.1 to 0.2)	0.217
Mean (SD) maternal MUAC (cm) at 1 month after delivery										
Adjusted for the timing of measurement and MUAC at enrollment	24.8 (1.3)	24.9 (1.3)	25.1 (1.4)	0.046	0.1 (−0.1 to 0.3)	0.414	0.2 (0.0 to 0.4)	0.015	0.2 (0.0 to 0.3)	0.109
Adjusted for the timing of measurement and MUAC, malaria, gravidity, and adolescence at enrollment	24.8 (1.3)	24.9 (1.3)	25.1 (1.4)	0.069	0.1 (−0.1 to 0.3)	0.415	0.2 (0.0 to 0.4)	0.023	0.1 (0.0 to 0.3)	0.145
Mean (SD) maternal weight (kg) at 1 month after delivery										
Adjusted for the timing of measurement and weight at enrollment	51.0 (3.0)	51.1 (2.9)	51.4 (2.9)	0.102	0.2 (−0.3 to 0.6)	0.443	0.4 (0.0 to 0.9)	0.035	0.3 (−0.1 to 0.7)	0.184
Adjusted for the timing of measurement and weight, malaria, gravidity, and adolescence at enrollment	51.0 (3.0)	51.1 (2.8)	51.4 (2.9)	0.193	0.1 (−0.3 to 0.6)	0.491	0.4 (0.0 to 0.8)	0.072	0.2 (−0.2 to 0.6)	0.270
Mean (SD) maternal BMI (kg/m^2^) at 1 month after delivery										
Adjusted for the timing of measurement and BMI at enrollment	21.2 (1.2)	21.2 (1.2)	21.3 (1.2)	0.115	0.1 (−0.1 to 0.3)	0.356	0.2 (0.0 to 0.4)	0.038	0.1 (−0.1 to 0.3)	0.256
Adjusted for the timing of measurement and BMI, malaria, gravidity, and adolescence at enrollment	21.2 (1.2)	21.2 (1.2)	21.3 (1.2)	0.229	0.1 (−0.1 to 0.2)	0.402	0.1 (0.0 to 0.3)	0.086	0.1 (−0.1 to 0.2)	0.383

AZI-SP = azithromycin and sulfadoxine–pyrimethamine; BMI = body mass index; gw = gestational weeks; MUAC = mid-upper arm circumference; SP = sulfadoxine–pyrimethamine.

Sensitivity analyses that took into account the clustering of participants who participated in the trial twice yielded similar results to the main analyses (Supplemental Table 1). There was no difference between the groups in the prevalence of facial, hand, or pitting edema (overall prevalence 1.0%) nor in the prevalence of self-reported chronic diarrhea (overall prevalence 0.2%) during pregnancy follow-up (data not shown).

## DISCUSSION

The main aim of this secondary analysis was to analyze the effect of an intensified IPTp regimen with monthly SP, with or without azithromycin, on maternal weight gain and anemia during pregnancy and at 1 month after delivery, compared with the standard regimen at the time the study was implemented (two SP doses). The same analyses were also undertaken between the group that received the current standard regimen of monthly SP and those who received monthly SP and two doses of azithromycin. In our study sample, the participants who received IPTp with monthly SP and two doses of azithromycin gained more weight during the second and third trimester of pregnancy than the control or monthly SP group participants. This difference was especially prominent among HIV-positive participants, who had higher weekly GWG both in the AZI-SP and monthly SP groups, compared with the controls, whereas the difference between the AZI-SP and monthly SP groups was not significant. Among HIV-negative participants, a difference was only noted between the AZI-SP and monthly SP groups. Hemoglobin and the prevalence of anemia during or after pregnancy did not differ between the study groups.

The strengths of our study include the large sample size, broad inclusion criteria, random group allocation, comprehensive follow-up, partial use of placebo control, and blinding of the outcome assessors. The weaknesses include the lack of information on prepregnancy weight, dietary intake, physical activity, or other energy use of participants, all of which could have an effect on GWG. However, we have no reason to believe that these would have differed between the study groups. Furthermore, we only tested for a limited number of infections, none of which were intestinal bacteria, viruses, or parasites, which can increase the risk of malnutrition, and we did not analyze the intake of medication against such agents (e.g., albendazole) or measure any inflammatory markers. As a proxy, we analyzed the prevalence of chronic diarrhea, but its prevalence overall was low, and there were no differences between the study groups, nor were there differences in the safety of the source of drinking water or adequacy of sanitation. It should be noted that although the standard IPTp regimen has changed since the implementation of this study from two to at least three doses of IPTp-SP during pregnancy, and in the current situation, it would be ideal to use the monthly SP group as the control group in the analyses, the study was not powered to test formal hypotheses between the monthly SP and AZI-SP groups. In addition, the validity and magnitude of the results among HIV-positive pregnant women might be different during the current situation of widely available ART and the use of cotrimoxazole prophylaxis, which cannot be administered concurrently with SP.

Thus, we consider the probabilities of bias, random error, and type I error low and believe that the sample findings are reliable and representative, meaning that in the study area, GWG can be improved by providing monthly SP and two doses of azithromycin to pregnant women, with the largest effect noted when comparing the two intervention groups to the control group among those who were HIV-positive. Higher MUAC in the AZI-SP group than in the control group 1 month after delivery suggests that the intervention might also benefit women during the postnatal period. Monthly SP alone may have a small positive effect on the outcomes, but the higher likelihood of random error makes population inference less conclusive for this regimen.

The average weekly GWG in this rural Malawian population was very low during the last two trimesters (256 g/week), compared with the international recommendation (420 g/week for normal-weight women), yet similar to earlier observations (240–259 g/week) from the study area.^33^^,^^37^^,^^38^ Two other randomized controlled trials using IPTp with SP and azithromycin reported results on GWG and anemia, but the intervention and/or control groups in these trials differed from ours.^28^^,^^39^^,^^40^ The APPLe Study, which was also conducted in rural Malawi, found no difference in maternal anemia during pregnancy between participants who received SP twice or both SP and azithromycin (1 g) twice.^39^ This study did not report GWG by study group.^40^

The study conducted in PNG found that pregnant women who received a median of three treatment courses of SP (same dose as in our trial) and a higher dose of azithromycin (4 g) gained, on average, 419 g/week, which was 58 g (95% CI 26 to 90; *P* <0.001) more than women who received a single treatment course of SP and chloroquine (450–600 g for 3 days) at their first antenatal visit.^28^ However, a different method and adjustments were used to calculate the weekly GWG in this study, compared with ours; therefore, the absolute values are not directly comparable.^28^ There was no difference in the prevalence of anemia or mean Hb at delivery between the study groups in the PNG study.^29^ The low prevalence of HIV among the participants of that study prevented analyses stratified by HIV status.^29^ Similar to our study, the study conducted in PNG found higher maternal MUAC at postnatal visit in the group that received azithromycin.^28^ In addition, just as in our study, the group that received azithromycin in the PNG study had a lower prevalence of maternal malaria and preterm delivery, as well as higher mean birthweight, whereas the APPLe study conducted in Malawi reported no differences in these outcomes between the study groups.^29^^,^^30^^,^^32^^,^^40^

A possible explanation for higher GWG in the group receiving azithromycin in our study and the PNG study is the lower burden of infections in this group, compared with the controls. Both studies reported a lower prevalence of malaria at delivery, and ours also reported a lower prevalence of malaria during pregnancy, compared with the controls.^29^^–^^31^ However, in our study, the participants in the monthly SP group also had a lower prevalence of malaria during pregnancy than those in the control group, whereas there was no difference in GWG or birth outcomes between the monthly SP and control groups.^30^^,^^31^ Thus, additional factors likely explain the improved GWG and other positive outcomes in the AZI-SP group, such as non-malarial effects similar to those attributed to SP.^41^^,^^42^ In our study, the participants in the AZI-SP group had a lower prevalence of trichomonas at one month after delivery than the control group participants, whereas there was no difference in the prevalence of trichomonas between the monthly SP and control groups, while the PNG study noted lower carriage of *Neisseria gonorrhoeae* among participants who received azithromycin.^29^^,^^30^ There might have been additional infective agents, including enteric pathogens, for which neither study tested but against which azithromycin is effective as a broad-spectrum antibiotic.^26^^,^^43^^–^^45^

Other possible explanations for higher maternal weight gain in the AZI-SP group include azithromycin’s effects on the maternal gut microbiome and azithromycin’s anti-inflammatory effect, which could reduce maternal energy expenditure and/or improve maternal dietary energy harvest.^20^^,^^46^^–^^48^ A secondary analysis of the study conducted in PNG suggested that azithromycin reduced inflammation among pregnant women, regardless of whether they had malaria.^47^ The study also found that biomarkers of inflammation and infection at the first antenatal visit and delivery were associated with adverse pregnancy outcomes in the arm that did not receive azithromycin.^47^ These findings support the hypothesis that some of the positive effects of azithromycin could be due to its anti-inflammatory effect.

## CONCLUSION

Our results and those of the study conducted in PNG suggest that preventive treatment of infections during pregnancy with azithromycin improves GWG, which is one possible gateway to improving pregnancy outcomes.^5^^,^^28^ In addition to having higher GWG and lower prevalence of both preterm delivery and LBW, the group that received azithromycin had higher maternal postnatal MUAC and improved infant growth until at least 1 month postpartum in both studies.^28^^–^^30^^,^^32^ Further follow-up in our study suggested that the AZI-SP intervention continued to benefit the children; they had lower post-neonatal mortality, higher developmental scores, and lower cumulative incidence of underweight at 5 years of age, as well as lower cumulative incidence of ever being stunted at ∼13 years of age, than children born to the control group.^49^^–^^51^ These wide-ranging possible benefits warrant future studies regarding the mechanisms by which azithromycin improves these outcomes, taking into account the current IPTp, HIV, and prophylactic treatment guidelines. In addition, future trials should closely monitor for changes in pathogen resistance to azithromycin, as well as possible longer-term adverse consequences of azithromycin for the infant.

## Supplemental Materials

10.4269/ajtmh.23-0829 Supplemental Materials

## References

[1] Victora CG Christian P Vidaletti LP Gatica-Domínguez G Menon P Black RE, 2021. Revisiting maternal and child undernutrition in low-income and middle-income countries: Variable progress towards an unfinished agenda. Lancet 397: 1388–1399. 33691094 10.1016/S0140-6736(21)00394-9 PMC7613170

[2] Christian P, 2018. Nutrition and maternal survival in low and middle income countries. Lammi-Keefe CJ Couch SC Kirwan JP. Handbook of Nutrition and Pregnancy. 2^nd^ ed. Cham, Switzerland: Springer Nature, 401–422.

[3] Lee SE Talegawkar SA Merialdi M Caulfield LE, 2013. Dietary intakes of women during pregnancy in low- and middle-income countries. Public Health Nutr 16: 1340–1353. 23046556 10.1017/S1368980012004417 PMC10271363

[4] Independent Expert Group, 2021. 2021 Global Nutrition Report: The State of Global Nutrition. Available at: https://globalnutritionreport.org/reports/2021-global-nutrition-report/dataset-and-metadata/. Accessed February 11, 2022.

[5] Goldstein RF , 2017. Association of gestational weight gain with maternal and infant outcomes: A systematic review and meta-analysis. JAMA 317: 2207–2225. 28586887 10.1001/jama.2017.3635 PMC5815056

[6] Young MF Oaks BM Tandon S Martorell R Dewey KG Wendt AS, 2019. Maternal hemoglobin concentrations across pregnancy and maternal and child health: A systematic review and meta-analysis. Ann NY Acad Sci 1450: 47–68. 30994929 10.1111/nyas.14093 PMC6767572

[7] Perumal N ; GWG Pooling Project Consortium, 2023. Suboptimal gestational weight gain and neonatal outcomes in low and middle income countries: Individual participant data meta-analysis. BMJ 382: e072249. 37734757 10.1136/bmj-2022-072249 PMC10512803

[8] Katz J ; CHERG Small-for-Gestational-Age-Preterm Birth Working Group, 2013. Mortality risk in preterm and small-for-gestational-age infants in low-income and middle-income countries: A pooled country analysis. Lancet 382: 417–425. 23746775 10.1016/S0140-6736(13)60993-9 PMC3796350

[9] Stein AD ; Consortium of Health-Orientated Research in Transitioning Societies (COHORTS) investigators, 2013. Birth status, child growth, and adult outcomes in low- and middle-income countries. J Pediatr 163: 1740–1746.e4. 24064150 10.1016/j.jpeds.2013.08.012 PMC3849851

[10] Blencowe H Cousens S Chou D Oestergaard M Say L Moller AB Kinney M Lawn J; Born Too Soon Preterm Birth Action Group, 2013. Born too soon: The global epidemiology of 15 million preterm births. Reprod Health 10 *(*Suppl 1*):* S2. 24625129 10.1186/1742-4755-10-S1-S2 PMC3828585

[11] Crump C, 2020. Preterm birth and mortality in adulthood: A systematic review. J Perinatol 40: 833–843. 31767981 10.1038/s41372-019-0563-y PMC7246174

[12] Victora CG Adair L Fall C Hallal PC Martorell R Richter L Singh Sachdev H; Maternal and Child Undernutrition Study Group, 2008. Maternal and child undernutrition: Consequences for adult health and human capital. Lancet 371: 340–357. 18206223 10.1016/S0140-6736(07)61692-4 PMC2258311

[13] Wang D Wang M Darling AM Perumal N Liu E Danaei G Fawzi WW, 2020. Gestational weight gain in low-income and middle-income countries: A modelling analysis using nationally representative data. BMJ Glob Health 5: e003423. 10.1136/bmjgh-2020-003423 PMC7661366 33177038

[14] Christian P Mullany LC Hurley KM Katz J Black RE, 2015. Nutrition and maternal, neonatal, and child health. Semin Perinatol 39: 361–372. 26166560 10.1053/j.semperi.2015.06.009

[15] Hambidge KM ; Women First Preconception Trial Study Group, 2019. A multicountry randomized controlled trial of comprehensive maternal nutrition supplementation initiated before conception: The Women First trial. Am J Clin Nutr 109: 457–469. 30721941 10.1093/ajcn/nqy228 PMC6367966

[16] Kinshella M-LW Omar S Scherbinsky K Vidler M Magee LA von Dadelszen P Moore SE Elango R; The PRECISE Conceptual Framework Working Group, 2021. Effects of maternal nutritional supplements and dietary interventions on placental complications: An umbrella review, meta-analysis and evidence map. Nutrients 13: 472. 33573262 10.3390/nu13020472 PMC7912620

[17] Liu E ; members of the GWG Pooling Project Consortium, 2022. Effects of prenatal nutritional supplements on gestational weight gain in low- and middle-income countries: A meta-analysis of individual participant data. Am J Clin Nutr 116: 1864–1876. 36130877 10.1093/ajcn/nqac259 PMC10843965

[18] Schaible UE Kaufmann SH, 2007. Malnutrition and infection: Complex mechanisms and global impacts. PLoS Med 4: e115. 17472433 10.1371/journal.pmed.0040115 PMC1858706

[19] Katona P Katona-Apte J, 2008. The interaction between nutrition and infection. Clin Infect Dis 46: 1582–1588. 18419494 10.1086/587658

[20] Raiten DJ Sakr Ashour FA Ross AC Meydani SN Dawson HD Stephensen CB Brabin BJ Suchdev PS van Ommen B; INSPIRE Consultative Group, 2015. Inflammation and nutritional science for programs/policies and interpretation of research evidence (INSPIRE). J Nutr 145: 1039S–1108S. 25833893 10.3945/jn.114.194571 PMC4448820

[21] White NJ, 2018. Anaemia and malaria. Malar J 17: 371. 30340592 10.1186/s12936-018-2509-9 PMC6194647

[22] Thompson JM Eick SM Dailey C Dale AP Mehta M Nair A Cordero JF Welton M, 2020. Relationship between pregnancy-associated malaria and adverse pregnancy outcomes: A systematic review and meta-analysis. J Trop Pediatr 66: 327–338. 31598714 10.1093/tropej/fmz068

[23] Saito M Briand V Min AM McGready R, 2020. Deleterious effects of malaria in pregnancy on the developing fetus: A review on prevention and treatment with antimalarial drugs. Lancet Child Adolesc Health 4: 761–774. 32946830 10.1016/S2352-4642(20)30099-7

[24] World Health Organization, 2021. World Malaria Report 2021. Geneva, Switzerland: WHO.

[25] Kayentao K , 2013. Intermittent preventive therapy for malaria during pregnancy using 2 vs 3 or more doses of sulfadoxine-pyrimethamine and risk of low birth weight in Africa: Systematic review and meta-analysis. JAMA 309: 594–604. 23403684 10.1001/jama.2012.216231 PMC4669677

[26] Pfizer Inc, 2021. Zithromax®. Highlights of Prescribing Information. Available at: https://labeling.pfizer.com/ShowLabeling.aspx?id=511. Accessed April 10, 2022.

[27] Burns AL , 2020. Retargeting azithromycin analogues to have dual-modality antimalarial activity. BMC Biol 18: 133. 32993629 10.1186/s12915-020-00859-4 PMC7526119

[28] Unger HW Wangnapi RA Ome-Kaius M Boeuf P Karl S Mueller I Rogerson SJ, 2016. Azithromycin-containing intermittent preventive treatment in pregnancy affects gestational weight gain, an important predictor of birthweight in Papua New Guinea—An exploratory analysis. Matern Child Nutr 12: 699–712. 26373537 10.1111/mcn.12215 PMC6860090

[29] Unger HW , 2015. Sulphadoxine-pyrimethamine plus azithromycin for the prevention of low birthweight in Papua New Guinea: A randomised controlled trial. BMC Med 13: 9. 25591391 10.1186/s12916-014-0258-3 PMC4305224

[30] Luntamo M Kulmala T Mbewe B Cheung YB Maleta K Ashorn P, 2010. Effect of repeated treatment of pregnant women with sulfadoxine-pyrimethamine and azithromycin on preterm delivery in Malawi: A randomized controlled trial. Am J Trop Med Hyg 83: 1212–1220. 21118924 10.4269/ajtmh.2010.10-0264 PMC2990034

[31] Luntamo M Rantala AM Meshnick SR Cheung YB Kulmala T Maleta K Ashorn P, 2012. The effect of monthly sulfadoxine-pyrimethamine, alone or with azithromycin, on PCR-diagnosed malaria at delivery: A randomized controlled trial. PLoS ONE 7: e41123. 22829919 10.1371/journal.pone.0041123 PMC3400634

[32] Luntamo M Kulmala T Cheung YB Maleta K Ashorn P, 2013. The effect of antenatal monthly sulphadoxine-pyrimethamine, alone or with azithromycin, on foetal and neonatal growth faltering in Malawi: A randomised controlled trial. Trop Med Int Health 18: 386–397. 23432801 10.1111/tmi.12074

[33] Kulmala T Vaahtera M Ndekha M Koivisto AM Cullinan T Salin ML Ashorn P, 2001. Gestational health and predictors of newborn weight amongst pregnant women in rural Malawi. Afr J Reprod Health 5: 99–108. 12471934

[34] Center for Social Research, Save the Children Federation USA, Malawi Ministry of Health and Population, MEASURE Evaluation, 2004. Avoiding Unwanted Pregnancy and Sexually Transmitted Infections: A Rural Malawi District Study. Chapel Hill, NC: MEASURE Evaluation.

[35] World Health Organization, 2023. Guidelines for Malaria. Geneva, Switzerland: WHO.

[36] Cheung YB, 2014. Statistical Analysis of Human Growth and Development. Boca Ration, FL: CRC Press.

[37] Rasmussen KM Yaktine AL; Committee to Reexamine IOM Pregnancy Weight Guidelines, Food and Nutrition Board, Board on Children, Youth and Families, Institute of Medicine, National Research Council, 2009. Weight Gain During Pregnancy: Reexamining the Guidelines. Washington, D.C.: The National Academies Press. 20669500

[38] Hartikainen H Maleta K Kulmala T Ashorn P, 2005. Seasonality of gestational weight gain and foetal growth in rural Malawi. East Afr Med J 82: 294–299. 16175780 10.4314/eamj.v82i6.9300

[39] van den Broek NR White SA Goodall M Ntonya C Kayira E Kafulafula G Neilson JP, 2009. The APPLe study: A randomized, community-based, placebo-controlled trial of azithromycin for the prevention of preterm birth, with meta-analysis. PLoS Med 6: e1000191. 19956761 10.1371/journal.pmed.1000191 PMC2776277

[40] van den Broek NR Jean-Baptiste R Neilson JP, 2014. Factors associated with preterm, early preterm and late preterm birth in Malawi. PLoS One 9: e90128. 24595186 10.1371/journal.pone.0090128 PMC3940843

[41] Roh ME , 2020. Overall, anti-malarial, and non-malarial effect of intermittent preventive treatment during pregnancy with sulfadoxine-pyrimethamine on birthweight: A mediation analysis. Lancet Glob Health 8: e942–e953. 32562650 10.1016/S2214-109X(20)30119-4 PMC7303957

[42] Waltmann A , 2022. The positive effect of malaria IPTp-SP on birthweight is mediated by gestational weight gain but modifiable by maternal carriage of enteric pathogens. EBioMedicine 77: 103871. 35217408 10.1016/j.ebiom.2022.103871 PMC8866062

[43] Capan M Mombo-Ngoma G Makristathis A Ramharter M, 2010. Anti-bacterial activity of intermittent preventive treatment of malaria in pregnancy: Comparative in vitro study of sulphadoxine-pyrimethamine, mefloquine, and azithromycin. Malar J 9: 303. 21029476 10.1186/1475-2875-9-303 PMC2984572

[44] Gordillo ME Singh KV Murray BE, 1993. In vitro activity of azithromycin against bacterial enteric pathogens. Antimicrob Agents Chemother 37: 1203–1205. 8390813 10.1128/aac.37.5.1203 PMC187935

[45] Riddle MS , 2017. Guidelines for the prevention and treatment of travelers’ diarrhea: A graded expert panel report. J Travel Med 24: S57–S74. 10.1093/jtm/tax026 PMC5731448 28521004

[46] Angelakis E Merhej V Raoult D, 2013. Related actions of probiotics and antibiotics on gut microbiota and weight modification. Lancet Infect Dis 13: 889–899. 24070562 10.1016/S1473-3099(13)70179-8

[47] Unger HW , 2019. Sulphadoxine-pyrimethamine plus azithromycin may improve birth outcomes through impacts on inflammation and placental angiogenesis independent of malarial infection. Sci Rep 9: 2260. 30783215 10.1038/s41598-019-38821-2 PMC6381158

[48] Zimmermann P Ziesenitz VC Curtis N Ritz N, 2018. The immunomodulatory effects of macrolides—A systematic review of the underlying mechanisms. Front Immunol 9: 302. 29593707 10.3389/fimmu.2018.00302 PMC5859047

[49] Hallamaa L Cheung YB Maleta K Luntamo M Ashorn U Gladstone M Kulmala T Mangani C Ashorn P, 2018. Child health outcomes after presumptive infection treatment in pregnant women: A randomized trial. Pediatrics 141: e20172459. 29472491 10.1542/peds.2017-2459

[50] Hallamaa L Cheung YB Luntamo M Ashorn U Kulmala T Mangani C Ashorn P, 2019. The impact of maternal antenatal treatment with two doses of azithromycin and monthly sulphadoxine-pyrimethamine on child weight, mid-upper arm circumference and head circumference: A randomized controlled trial. PLoS One 14: e0216536. 31063503 10.1371/journal.pone.0216536 PMC6504037

[51] Videman K Hallamaa L Heimonen O Mangani C Luntamo M Maleta K Ashorn P Ashorn U, 2022. Child growth and neurodevelopment after maternal antenatal antibiotic treatment. Arch Dis Child 107: 323–328. 34479861 10.1136/archdischild-2021-322043 PMC8938655

