# Supplementary material for: Effect of Antenatal Monthly Sulfadoxine–Pyrimethamine, Alone or with Azithromycin, on Gestational Weight Gain and Anemia during Pregnancy and One Month Postpartum in Malawi: A Randomized Controlled Trial Secondary Analysis
Source: Am J Trop Med Hyg. 2025 Feb 4;112(4):931–41. doi: 10.4269/ajtmh.23-0829 (PMC11965741; doi:10.4269/ajtmh.23-0829)
Supplement: Supplemental Materials [file tpmd230829.SD1.pdf]

SUPPLEMENTAL TABLE 1

Sensitivity analyses allowing clustering of 48 individuals who participated in the trial twice with consecutive pregnancies

Outcome	Three-group *P*-value	Comparison Between Monthly SP and Control Group		Comparison Between AZI-SP and Control Group		Comparison Between AZI-SP and Monthly SP Group	
		Mean Difference or RR (95% CI)	*P*-value	Mean Difference or RR (95% CI)	*P*-value	Mean Difference or RR (95% CI)	*P*-value
Mean (SD) GWG (g/week) from enrollment to delivery							
Unadjusted, allowing clustering	0.005	3 (-13 to 19)	0.685	24 (8 to 40)	0.003	21 (5 to 37)	0.010
Mean (SD) Hb (g/l) at 28.00-33.99 gw							
Adjusted for the timing of measurement and Hb at enrollment, allowing clustering	0.063	1 (-1 to 3)	0.282	2 (0 to 4)	0.019	1 (-1 to 3)	0.194
Mean (SD) maternal Hb (g/l) at 1 month after delivery							
Adjusted for the timing of measurement and Hb at enrollment, allowing clustering	0.223	1 (-2 to 3)	0.511	2 (0 to 4)	0.085	1 (-1 to 4)	0.323
Mean (SD) MUAC (cm) at 28.00-33.99 gw							
Adjusted for the timing of measurement and MUAC at enrollment, allowing clustering	0.089	0.1 (-0.1 to 0.2)	0.436	0.2 (0.0 to 0.3)	0.030	0.1 (0.0 to 0.2)	0.173
Mean (SD) maternal MUAC (cm) at 1 month after delivery							
Adjusted for the timing of measurement and MUAC at enrollment, allowing clustering	0.051	0.1 (-0.1 to 0.3)	0.404	0.2 (0.0 to 0.4)	0.016	0.2 (0.0 to 0.3)	0.108
Mean (SD) maternal weight (kg) at 1 month after delivery							
Adjusted for the timing of measurement and weight at enrollment, allowing clustering	0.104	0.2 (-0.3 to 0.6)	0.444	0.4 (0.0 to 0.9)	0.036	0.3 (-0.1 to 0.7)	0.180
Mean (SD) maternal BMI (kg/m^2) at 1 month after delivery							
Adjusted for the timing of measurement and BMI at enrollment, allowing clustering	0.117	0.1 (-0.1 to 0.3)	0.358	0.2 (0.0 to 0.4)	0.039	0.1 (-0.1 to 0.3)	0.250

SP = sulfadoxine–pyrimethamine; AZI-SP = azithromycin and sulfadoxine–pyrimethamine; GWG = gestational weight gain; Hb = hemoglobin; gw = gestational weeks; MUAC = mid-upper arm circumference; BMI = body mass index.
